# Primary Pulmonary Echinococcosis in the United States: A Case Report and Review of the Literature

**DOI:** 10.7759/cureus.55591

**Published:** 2024-03-05

**Authors:** Nathan Zhang, Neil K Vuppala, Colton P Boney, Justin Moon, Ryan Liengswangwong, Heong Jin C Ahn, Albert Tine, Todd J Kendall

**Affiliations:** 1 Medicine, Alabama College of Osteopathic Medicine, Dothan, USA; 2 Medicine, Alabama College of Osteopathic Medicine, Huntsville, USA; 3 Internal Medicine, Alabama College of Osteopathic Medicine, Dothan, USA; 4 Research, Alabama College of Osteopathic Medicine, Dothan, USA; 5 Pathology, Ascension Providence Hospital, Mobile, USA

**Keywords:** role of imaging studies, helminth infection, infectious and parasitic diseases, thoracic surgeries, lung pathology, thoracic radiology, pulmonary hydatid cyst, echinococcosis granulosus

## Abstract

We depict a unique case of a 34-year-old woman who presents to the emergency department with complaints of dyspnea and chest pain for the past month. A chest x-ray (CXR) from an earlier urgent care visit was concerning for large fluid opacity in the left lung and follow-up imaging revealed a cystic mass suspicious of a pulmonary cystic abscess. The patient underwent complete lobectomy and resection. Post-surgical biopsy confirmed pulmonary hydatid cystic mass and signs of rupture or seeding to liver tissue. The patient was discharged with adjuvant therapy and recommended imaging follow-up for the next decade. The diagnosis, treatment, and maintenance guidelines are discussed in this report which reveals controversy between experts given the lack of complete literature regarding echinococcosis. Our purpose in putting forward this case is to present a rare diagnosis of pulmonary echinococcosis in the United States and to emphasize the importance of early imaging and diagnosis to prevent cystic rupture and secondary organ dissemination.

## Introduction

Echinococcosis, caused by the Taeniidae family of tapeworms, is a parasitic infection commonly manifesting as cystic or alveolar echinococcosis through parasitism from Echinococcus granulosus and Echinococcus multilocularis, respectively. Data on the tapeworm has been incomplete for the last several decades, but recent studies estimate a global incidence of over >200,000 new cases a year, and are endemic to regions such as Eastern Asia, South America, Africa, and the Mediterranean [1]. The echinococcus life cycle includes an intermediate wild-life and livestock host with fecal shedding of tapeworm eggs after initial infection of the small intestine. Humans become hosts during accidental ingestion of the eggs, most commonly after being exposed to domesticated dogs. Cystic echinococcosis, also known as hydatid cysts (HC), occurs after oncosphere migration through the small intestine. From the small intestine, these cysts can develop in various soft tissues and organ systems. The liver is the most common site for HC but pulmonary involvement and less commonly, extrapulmonary involvement are the second most common in adults (10%-30%) and the most common in children [2].

Diagnosis of initial infection can be done through various approaches including serology or imaging. Many modalities are appropriate including ultrasonography (US), computed tomography (CT), chest x-ray (CXR) and magnetic resonance imaging (MRI). Many pulmonary echinococcosis cases are incidentally discovered in CXR and highlight the asymptomatic nature of the infection, delaying diagnosis in many cases until significant mass effect or cystic rupture [2-4]. The primary treatment for echinococcosis involves surgical resection of the cysts resulting in an excellent prognosis for the majority of patients. However, there have been cases of pulmonary recurrence despite complete surgical lobectomy suggesting recalcitrant seeding and an aggressive clinical course of pulmonary involvement [5,6]. There is minimal literature and systematic review of North American echinococcus infections. In this report, we explore a case of a young woman with HC in the United States to better understand the role of imaging in pulmonary echinococcosis.

## Case presentation

**Figure 1:**
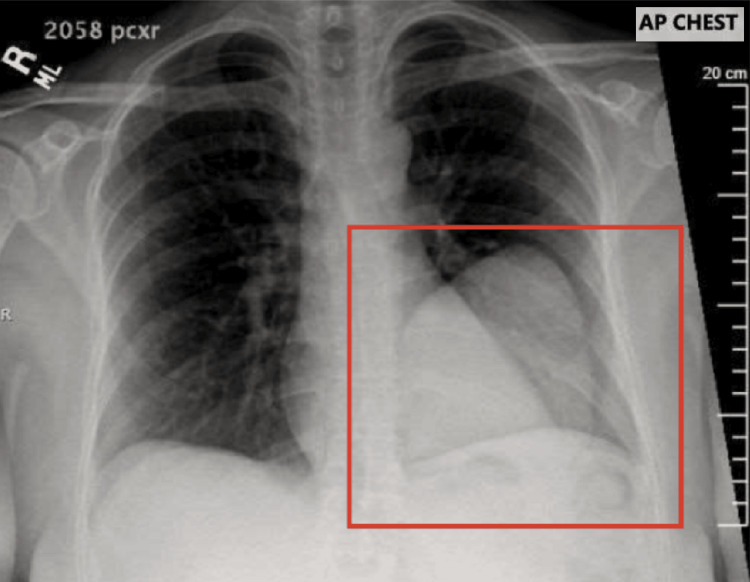
Chest x-ray with 9 cm cystic mass in the lower-left lobe

A 34-year-old Middle Eastern female presented to the emergency department with excessive coughing, mild dyspnea, cough, and left-sided chest pain for the past month. The patient had been referred from the urgent care due to concerns about fluid collection on the left lung on prior CXR. She had a history of well-controlled hypothyroidism and iron deficiency anemia with no significant surgical history aside from uncomplicated uterine myomectomy. She has no history of intravenous or illicit drug use and is a non-smoker and non-drinker. She immigrated from the Middle East where her family’s occupation was livestock-herding including sheep and cattle. Home medications included ferrous sulfate, levothyroxine, and docusate sodium. At the time of presentation, she denied any recent illness, respiratory infection, or sick contacts. She was 10 months postpartum and breastfeeding and has never had any imaging procedures done. Initial work-up included CXR completed shortly after admission, which revealed a 10cm x 8cm opacity in the left lower lobe (Figure 1).

**Figure 2:**
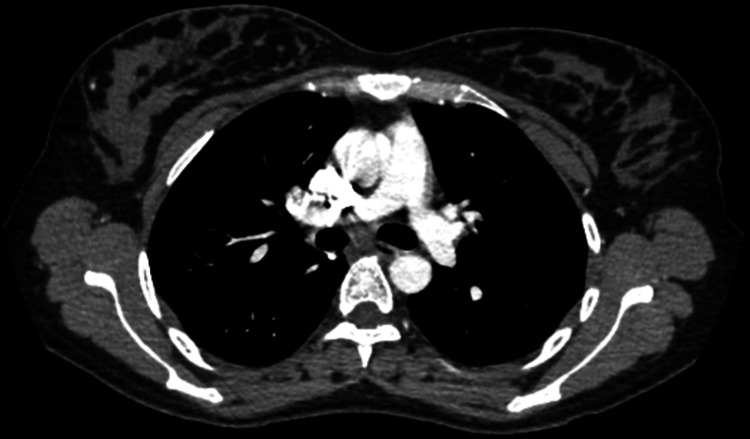
Axial CTA of PE workup with no apparent filling defects at the level of the pulmonary trunk bifurcation. There is some mild streak artefact from hyperdense contrast in the SVC. CTA - computed tomography angiography; SVC - superior vena cava

At the end of hospital day 1, CT angiography (CTA) of the chest (Figure 2) was also ordered to rule out symptomatic concerns of pulmonary embolism (PE) but no significant filling defects were observed in the larger pulmonary vessels decreasing the suspicion of symptomatic PE.

**Figure 3:**
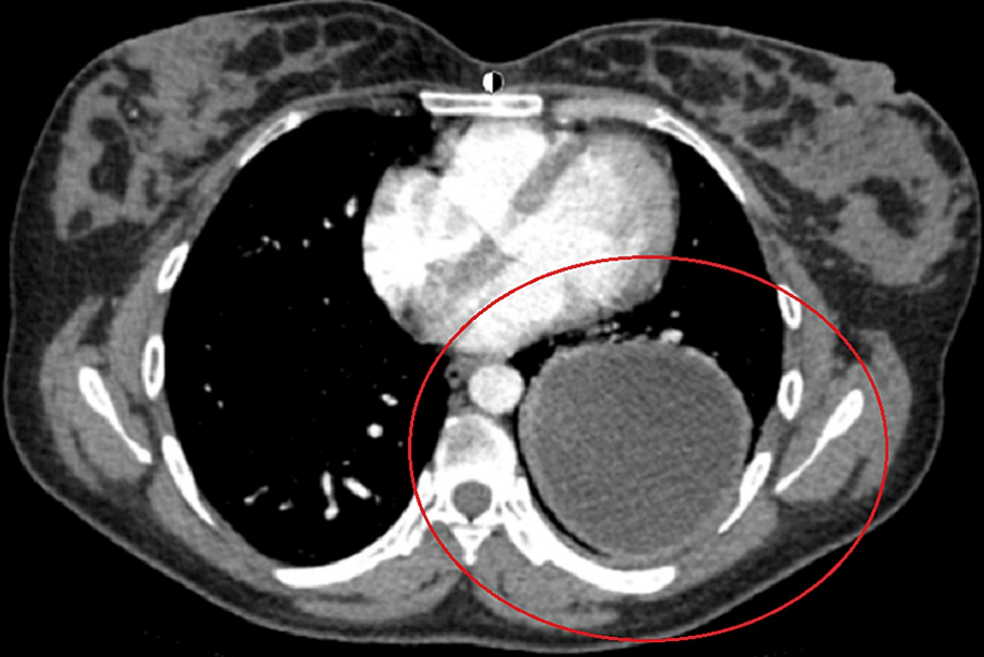
Axial CTA with a large fluid density and well-circumscribed, homogenous margins in the right posterior lung. CTA - computed tomography angiography

After reviewing the CTA, it was then revealed that there was a hypodense abnormality with characteristic features of a simple pulmonary cyst (Figure 3).

Additional lab work included complete blood count (CBC), comprehensive metabolic panel (CMP), and quantitative beta-hCG. Aminotransferase and Alkaline Phosphatase elevations were present with the remaining admission blood work and vital signs within normal limits (Table 1).

**Table 1:** Relevant labwork including complete blood count, comprehensive metabolic panel, liver function tests, D-Dimer, and β-hCG.

Lab	Patient value	Normal value
White blood cells	7.3 x 10^9^/L	4.5-11 x 10^9^/L
Neutrophils	60.40%	40%-60%
Eosinophils	1.20%	1%-3%
Lymphocytes	30.30%	25%-33%
BUN	16 mg/dl	7-18 mg/dL
Creatinine	1.1 mg/dl	0.6-1.2 mg/dL
Hemoglobin	12.9 g/dl	12-16 g/dL
Hematocrit	38.60%	36%-46%
Alanine aminotransferase (ALT)	65 U/mL	10-40 U/L
Aspartate aminotransferase (AST)	39 U/L	12-38 U/L
Alkaline Phosphatase	130 U/L	25-100 U/L
Bilirubin, Total	0.3 mg/dl	0.1-1.0 mg/dL
Beta-human Chorionic Gonadotropin (β-hCG)	<2.43 IU/L	<5 IU/L for non-gestational
D-Dimer	122 ng/mL	<250 ng/mL

On hospital day 2, Pulmonology was consulted for the abnormal CT images from the day prior. A working differential for infectious lobar consolidation was considered and Empiric IV Piperacillin-Tazobactam was initiated for a possible atypical, afebrile, pulmonary cystic abscess.

During hospital day 6, Thoracic Surgery was consulted due to repeat imaging and clinical symptoms without significant change. Based on the earlier CTA, the radiologist determined that CT-guided aspiration would carry a significant risk of spilling and the surgical team ultimately decided on a complete left lower lobe lobectomy for additional diagnostic evaluation. Cyst aspiration and evacuation proceeded without any gross evidence of purulent discharge or dissemination to surrounding lung parenchyma. Samples of the resected lung were sent for further analysis given the patient's geographic history. Post-surgical pathology of the clear cystic fluid was determined to be Echinococcus given the clinical history despite similar appearance hooklets seen in other members of the Taeniidae family (including T. Solium). Cytologic analysis of the left inferior pulmonary ligament lymph node biopsy was performed in tandem, unraveling mild anthracosis and benign lymphoid hyperplasia without malignancy. Notably, the echinococcus antibody serology returned negative, posing additional questions about the test's diagnostic sensitivity and utility in place of alternative diagnostic markers. The patient was discharged with Albendazole which treats both E. granulosus and E. multilocularis and recommended serial follow-up CXR for the next 10 years. She has not attended any post-excision follow-up observation with pathology, pulmonology, or surgery to monitor for recurrence.

## Discussion

While prevalence is higher in Asian and African countries, echinococcosis proves to be exceedingly rare in the United States, possibly due to the availability of early, inexpensive imaging [7]. Primary infection usually occurs in childhood through a sub-clinical phase reinforcing the need for early radiographic detection to prevent sequelae. Cough tends to be the most common symptom and recent studies suggest this can occur in up to 79% of infected adults and 93% of infected children [5]. Although serology has some value as a confirmatory approach, initial imaging is the diagnostic gold standard given the relatively high false negative rates of serology with some studies showing failure upwards of 50% in pulmonary involvement. Serology may have a more definitive role in identifying hepatic cysts (85% hepatic seropositivity) in adults compared to predominantly pulmonary seeding in children [5,8]. Likewise, laboratory markers of echinococcus are not reliable for initial diagnosis, as is the case with our patient. The expected leukocytosis and eosinophilia are often absent with peripheral eosinophilia occurring as sparingly as <25% of infected patients unless significant cyst rupture or dissemination occurs [5].

**Figure 4:**
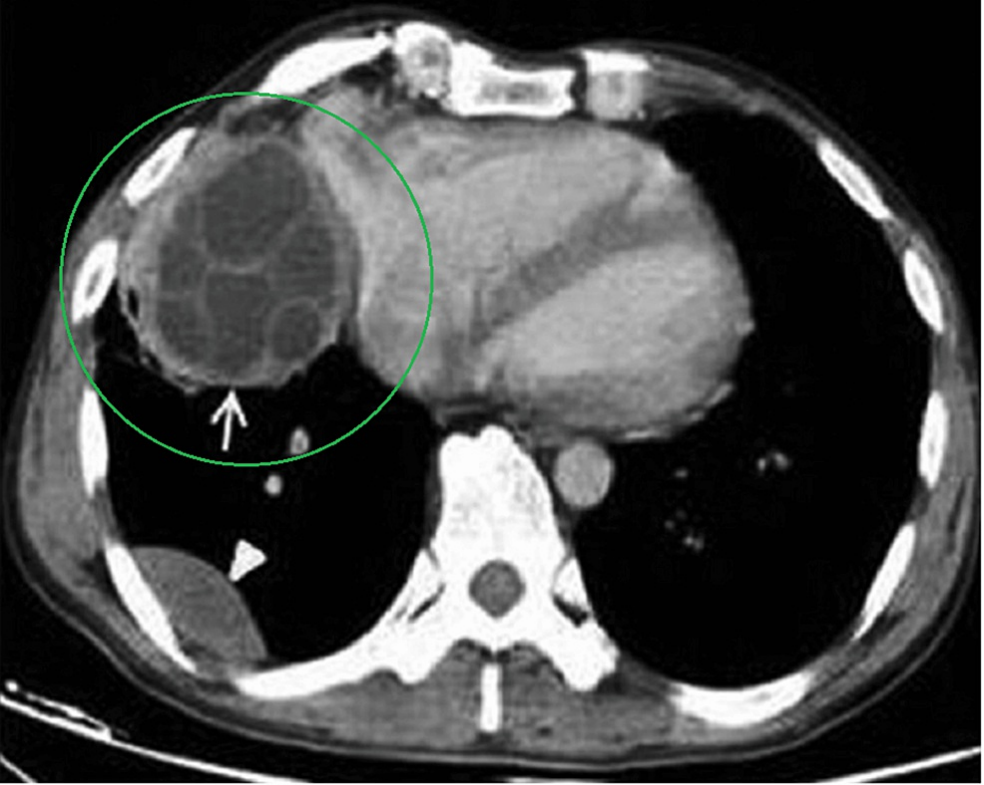
Example of an axial chest CT with contrast in a separate patient. This particular example shows cystic involvement in right cardiophrenic angle. Although these are different patients, the cysts appear similarly on both CTs with a unilocular, well-demarcated fluid density. Internal septations are present on this example due to multiple daughter cysts. Reference [8]

Of the many imaging modalities commonly used, US and X-ray tend to be more accessible, but CT has the highest sensitivity (95%+) and is the gold standard for determining the size, number, and location of HC caused by primary infection [9,10]. In comparison to portable US, several studies compared the diagnostic benefits of a CT approach including better identification of minor calcifications and gas contents within cysts [4,10]. On CT imaging, non-complex cysts are unilocular, well-demarcated, hypo-dense masses that present with or without hydatid sand and septation depending on the presence of daughter cysts (Figure 4).

On MRI, HC appear similarly with well-demarcated fluid hypo-intensity collections where MRI is preferred for better analysis of non-simple cysts with additional brood capsules or daughter cysts [9]. These imaging features of Pulmonary HC include a broad differential from bronchogenic cyst, hematoma, large granuloma, pulmonary abscess, and pulmonary malignancies (including primary lung carcinoma and sarcoma, mesothelioma, and lung metastasis, especially for multifocal HCs). The lack of systemic symptoms such as fatigue, fever, and weight loss. In addition, serial CT or MRI can help determine the extent of cyst calcification progression and secondary pulmonary complications including atelectasis, bronchiectasis, mediastinal lymphadenopathy, pleural thickening or effusion. Rarer pulmonary complications include hydatid aspiration and anaphylactic cough due to rupture as well as recurrent PE questioning the increased hyper-coagubility associated with surgical resection and hepatic infiltration of the parasite [3,11]. As a result, CTA has increased clinical importance in the exclusion of embolism given the overlapping symptoms with cyst rupture (i.e., thoraco-abdominal pain followed with or without hemoptysis, fever, and dyspnea) [5,6,8].

Treatment for E. granulosus can begin with a pharmacological-only approach with anti-parasitic benzimidazoles (Albendazole) for simple cysts with mild dissemination. On the other-hand, surgical excision guided by CT imaging has lower complications for the more aggressive E. multilocularis, or medically refractory cysts that have secondary risks of viscera compression [7]. In most scenarios, and as with our patient, surgery with adjunctive anti-parasitic drugs is definitive and aims to prevent larvae seeding due to complications (fluid spillage, cyst rupture, incomplete resection, etc.) [12]. While surgical removal is curative for active seeding, pulmonary cyst recurrence is increasingly common without anti-parasitics. Patients are recommended serial imaging for several years after primary resection to observe for refractory seeding and recurrence. This may be due to difficulty with complete drainage of exudates or persistent cystic fluid in the remnant pleural fluid [7,8]. One study in Turkey revealed that 11% of cysts recurred despite complete lobectomy and serial CXR follow-up is recommended from several years minimum, to upwards of the next decade [3,6]. Although imaging is the standard for both diagnosis and post-resection monitoring, additional non-maleficence concerns must be raised for minimizing radiation exposure. This is especially concerning in children who are more likely to have asymptomatic, and predominantly, pulmonary echinococcus involvement and brings a unique perspective on the risks of introducing early radiation exposure in children versus the benefits for early treatment exposure in areas with endemic echinococcus when immigrating to a country with relatively low incidence.

## Conclusions

This case draws attention to the infrequent occurrence of pulmonary echinococcus in the United States, underscoring the critical significance of timely diagnosis to prevent cystic rupture and the subsequent spread to other organs. The case incorporates the ongoing diagnostic difficulties with identifying pulmonary echinococcus in current literature and the increasingly pivotal role of imaging in recognizing and characterizing HC. Our case with a young adult with no prior imaging presents a unique case where the latent, asymptomatic childhood phase was not discovered on incidental imaging. With curative excision and early treatment, the prognosis is excellent. However, postoperative treatment guidelines vary in established literature, and several recommend increasingly long surveillance periods. Moreover, it is well established that recurrence is common in pulmonary echinococcosis despite the comprehensive surgical removal of cysts. Out-patient follow-up with periodic imaging for years post-excision is often advised but may be burdensome to patients who otherwise feel asymptomatic. This prolonged duration reinforces the persistent concern for a possible sinister cycle of reseeding and relapse.

This case reflects the unique clinical presentation, diagnostic challenges, and concurrent post-treatment hurdles associated with pulmonary echinococcosis in the United States. It advocates for heightened awareness among healthcare practitioners, emphasizing the need for early detection and vigilant postoperative monitoring to secure favorable patient outcomes. Advanced surgical excision and thorough resection are important to reduce the risk of recurrence. This case weighs the need for a more aggressive screening approach for patients who have historical geographic or occupational risk factors, despite the relatively low incidence in the current United States. The existing gaps in echinococcosis literature in North America further contribute to ongoing debates surrounding its identification and uncertain treatment duration.
